# Elevating the potential of CAR-T cell therapy in solid tumors: exploiting biomaterials-based delivery techniques

**DOI:** 10.3389/fbioe.2023.1320807

**Published:** 2024-01-18

**Authors:** Yuxiang Tang, Xiaoyu Yang, Hang Hu, Huiwen Jiang, Wei Xiong, Heng Mei, Yu Hu

**Affiliations:** ^1^ Tongji Medical College, Union Hospital, Institute of Hematology, Huazhong University of Science and Technology, Wuhan, China; ^2^ Hubei Clinical Medical Center of Cell Therapy for Neoplastic Disease, Wuhan, China; ^3^ Department of Pharmacy, Tongji Medical College, Union Hospital, Huazhong University of Science and Technology, Wuhan, China; ^4^ School of Pharmacy, ChangZhou University, Changzhou, China; ^5^ Wuhan Sian Medical Technology Co., Ltd., Wuhan, China

**Keywords:** chimeric antigen receptor-T, biomaterials, solid tumor, immunotherapy, nanobackpack

## Abstract

Chimeric antigen receptor (CAR) T cells exhibit promising progress in addressing hematologic malignancies. However, CAR-T therapy for solid tumors remains limited, with no FDA-approved CAR-T products available for clinical use at present. Primary reasons include insufficient infiltration, accumulation, tumor immunosuppression of the microenvironment, and related side effects. Single utilization of CAR-T cannot effectively overcome these unfavorable obstacles. A probable effective pathway to achieve a better CAR-T therapy effect would be to combine the benefits of biomaterials-based technology. In this article, comprehensive biomaterials strategies to break through these obstacles of CAR-T cell therapy at the tumor sites are summarized, encompassing the following aspects: 1) generating orthotopic CAR-T cells; 2) facilitating CAR-T cell trafficking; 3) stimulating CAR-T cell expansion and infiltration; 4) improving CAR-T cell activity and persistence; 5) reprogramming the immunosuppressive microenvironments. Additionally, future requirements for the development of this field, with a specific emphasis on promoting innovation and facilitating clinical translation, are thoroughly discussed.

## 1 Introduction

CAR-T therapy involves the genetic modification of T cells with synthetic chimeric antigen receptors (CARs) to specifically target combine the corresponding antigen expressed onto tumor cells, thus achieving precise tumor cell eradication. CARs are composed of three parts: extracellular single-chain variable fragment (scFv) for identifying tumors, a transmembrane fragment for transferring signals, and an intracellular signaling fragment for activating and costimulating T cells (Savoldo et al., 2011; Hay and Turtle, 2017). CAR-T is activated and kills target tumor cells once the scFv binds to tumor antigens (Depil et al., 2020). Innovative designs of CAR-T intracellular signaling domains have been developed in recent years. The first generation of CARs only used CD3ζ to recognize antigens expressed on tumor cells. The second generation of CARs further introduced a single costimulatory domain (e.g., 4-1BB, CD28) to enhance CAR-T cells’ fitness, whereas third-generation CARs generated more co-stimulatory domains aimed at expressing cytokines such as IL-15, IL-12, or cytokine receptors for stimulating CAR-T proliferation (Savoldo et al., 2011; Hay and Turtle, 2017). In conventional CAR-T cell clinical therapy, autologous T cells are collected from patients and genetically modified to create CAR-T cells. These CAR-T cells are expanded *in vitro* and then returned to the patient. Compared to chemotherapy drugs, CAR-T therapy has several advantages, including high efficacy, long-lasting effects, and active targeting. Furthermore, CAR-T cells have achieved significant progress in treating hematological malignancies (Neelapu et al., 2021). To date, both the CFDA and FDA have approved eight CAR-T products, including anti-BCMA CAR-T for multiple myeloma and anti-CD19 CAR-T for lymphomas.

**FIGURE 1:**
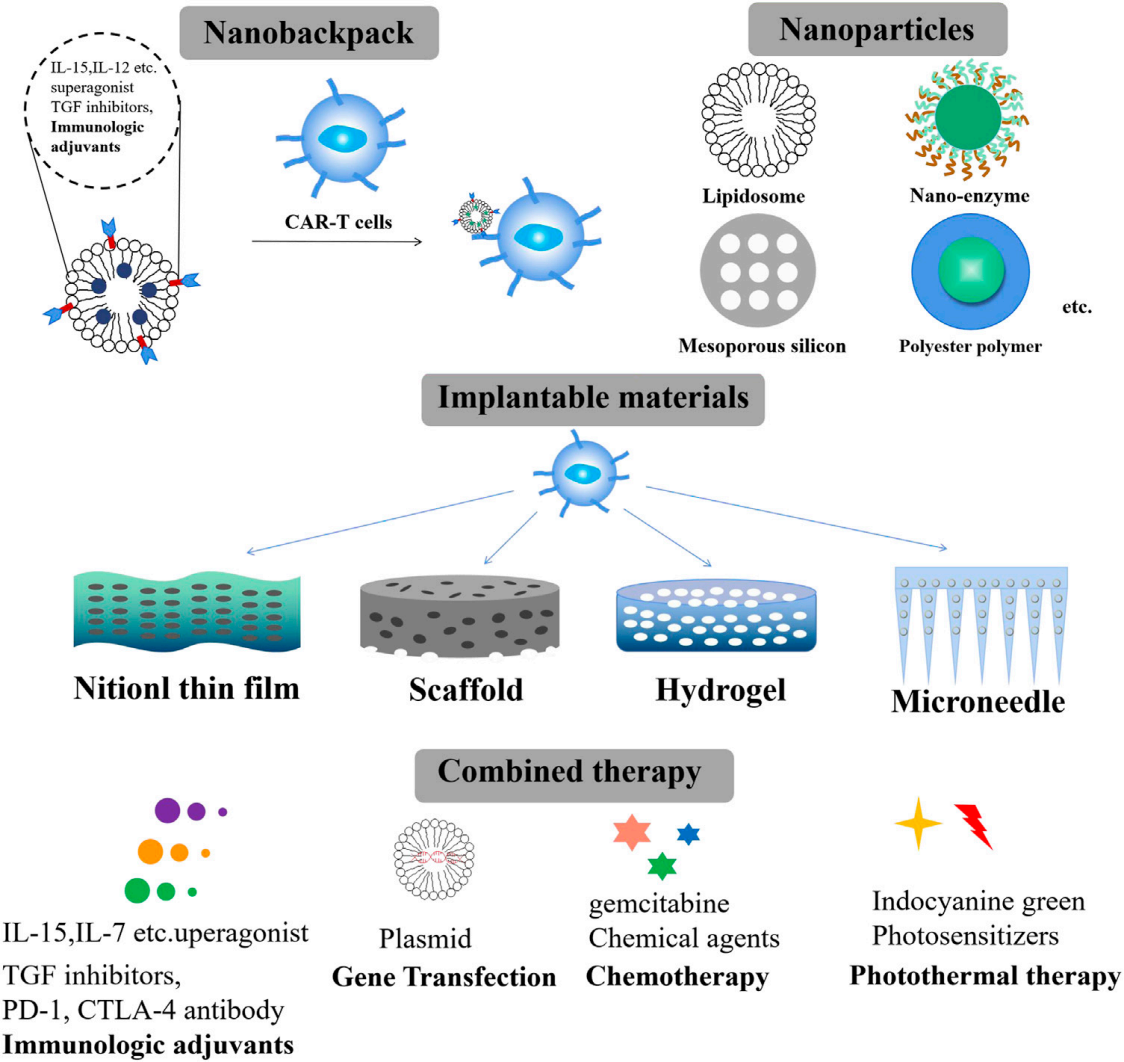
Different biomaterial strategies promote CAR-T functions toward solid tumors.

By considering the excellent clinical progression of CAR-T cells in the treatment of hematologic malignancies, more potent antigen-specific CARs (IL13Ra2, HER2, ALX) were designed to explore their application to solid tumors (Villa and Mischel, 2016; Hegde et al., 2016; Brown et al., 2013; Yi et al., 2018). However, their clinical results remain modest to date. Similar to the two types of GPC3 antigen-specific CAR-T cells for advanced hepatocellular carcinoma, 9 of 13 patients experienced CRS toxicity, with an overall survival rate of 10.5% at 3 years, 42% at 1 year, and 50.3% at 6 months (Shi et al., 2020). In the phase I clinical trial of HER2-CAR-T cells for pancreatic cancer, only 1 of 11 patients experienced partial remission after 4.5 months (Feng et al., 2018). Almost all solid tumor antigen-specific CAR-T cells experienced delay in the phase I clinical trial. Unlike treating hematologic malignancies, CAR-T therapy for solid tumors faces many obstacles. These obstacles could be attributed to the following (Table 1): 1) Trafficking and infiltration obstacles: solid tumors have a dense extracellular matrix (ECM) and the absence of specific tumor antigens. Chemokines secreted by solid tumors are usually not suitable for T cell infiltration (Bertrand et al., 2014; Wang et al., 2017); 2) Activation obstacle: Immune checkpoints such as PD-1, TIM-3, and CTLA-4 will be upregulated once CAR-T cells bind to tumor cells, thereby inhibiting immune responses (Long et al., 2015; Chmielewski and Abken, 2017; Adachi et al., 2018; Ma et al., 2020). IL-2 secreted by CAR-T cells may also stimulate the proliferation of T regulatory cells (Tregs) (Kofler et al., 2011); 3) Proliferation and survival obstacle: Solid tumor sites exhibit a hypoxic, acidic, and low-nutrient microenvironment (Dunn et al., 2019; Xue et al., 2022). As a result, the application of single CAR-T cells in treating solid tumors faces challenges in effectively overcoming these three obstacles, resulting in a slight therapeutic effect and high recurrence rates. Therefore, it is urgent to develop multiple therapeutic strategies for improving the tumor immunosuppressive microenvironment (TME) and synergizing CAR-T cells to promote their infiltration behaviors and therapeutic effect. To overcome these three obstacles, multiple biomaterials-based strategies have been employed to optimize the tumor microenvironment, target the delivery of CAR-T cells to the tumor site, and promote the infiltration of CAR-T cells. Various biomaterial strategies have made significant progress in the treatment of solid tumors. They can respond to vascular abnormalities, hypoxia, and acidic microenvironments of tumor sites, thereby facilitating the direct release of therapeutic agents into the tumor sites and improving the therapeutic effect. Therefore, adopting various combination biomaterial-based strategies for CAR-T treatment on solid tumors has become the focus of development in this field. Novel biomaterials should be flexible and expandable in structure, enabling their modifications on CAR-T cell surfaces or facilitating CAR-T combination therapy to enhance their function (Dunn et al., 2019; Han et al., 2022; Xue et al., 2022). As shown in Figure 1, three strategies based on biomaterials are employed in such cases. The first strategy is known as the nanobackpack approach. Nanobackpacks are conjugated onto CAR-T cells *in vitro* and then reinfused to facilitate CAR-T cell reach into the TME and controlled release of the loaded cargoes in the tumor site, thus enhancing CAR-T therapeutic effects. The second strategy involves the use of nanoparticles (NPs) as a key approach. To reverse the adverse tumor agents of the TME and enhance the proliferation, activation, and infiltration abilities of CAR-T cells, various NPs-based strategies, such as radiotherapy, chemotherapy, phototherapy, and blocking immune checkpoints, have been applied to remodel the TME, thus further facilitating CAR-T immunotherapy. The third strategy encompasses the utilization of implantable biomaterials as a crucial approach. In order to target traffic CAR-T cells to the tumor site and achieve enhanced immune effects, implantable biomaterials such as hydrogels, scaffolds, nitinol films, and microneedles are applied as CAR-T delivery systems. They can overcome physiological/physical obstacles and target traffic CAR-T cells to the tumor site, thus facilitating the proliferation and sustained characteristics of orthotopic CAR-T cells. In this review, we specifically summarized the principles of designating biomaterials and proposed biomaterials-based strategies that can facilitate the therapeutic effects of CAR-T cells. Moreover, we discussed the combination cases of CAR-T cells with biomaterials to overcome these three obstacles relevant to solid tumors.

**TABLE 1:** Solid tumors obstacles that hinder the therapy effects of CAR-T *in vivo*.

Category	Factors	Refs
Trafficking and Infiltration obstacles	compact tumor stroma cells tumor antigen loss	Bertrand et al. (2014), Wang et al. (2017)
Activation obstacles	immune checkpoints Treg cells block	Kofler et al. (2011), Long et al. (2015), Chmielewski and Abken (2017), Adachi et al. (2018), Ma et al. (2020)
Proliferation and Survival obstacles	slide acidic, low-nutrient, and hypoxia micro-environment	Dunn et al. (2019), Xue et al. (2022)

## 2 Designing biomaterials for CAR-T cells delivery

The design and fabrication of biomaterials should take into account a number of criteria relevant to immunomodulation. In general, all biomaterials should be designed to be biocompatible and biodegradable. It is crucial for the biomaterials to be inherently non-immunogenic to avoid the local inflammatory response (Kim et al., 2015). In this section, we will summarize the basic design principles for nanobackpacks (Cheung et al., 2018), NPs, and implantable biomaterials. We also highlight these recently developed biomaterials for CAR-T therapy.

### 2.1 Construction of nanobackpacks

**FIGURE 2:**
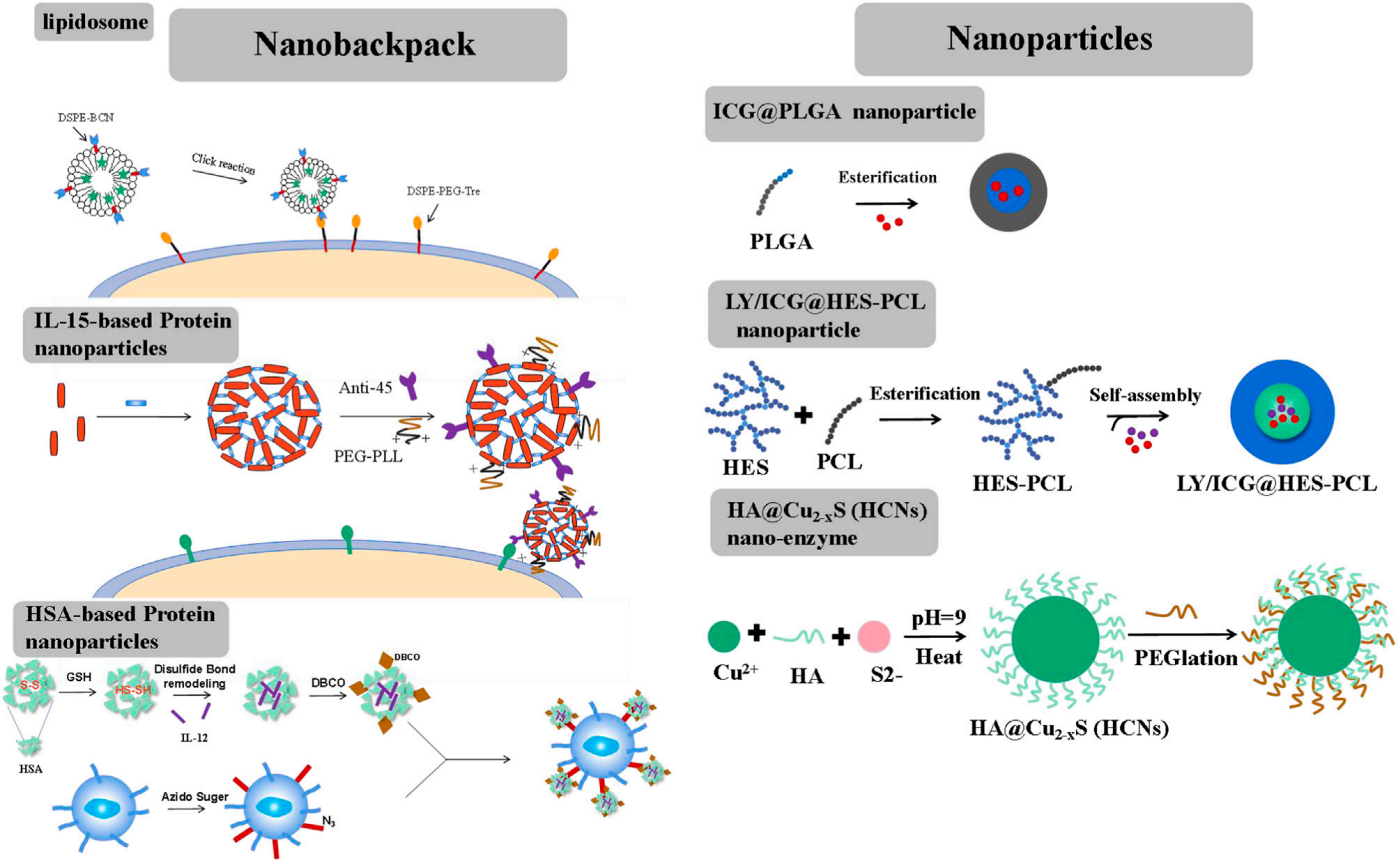
Different forms of nanobackpack and biomaterials promote CAR-T therapy toward solid tumors.

Nanobackpacks, engineering CAR-T cells with drug-loaded NPs, could achieve an enhanced therapeutic effect compared to single CAR-T therapy (Figure 2). By conjugating NPs onto CAR-T cells, they could be endowed with various enhanced functions *in vivo*, such as targeting, tracing, activation, and proliferation (Zheng et al., 2022). Nanobackpacks should be constructed at a nano-size (100–1000 nm) and be easily modified by CAR-T cells. Moreover, nanobackpacks could be stably anchored onto the cell surface without limiting CAR-T functions. It should be emphasized that the expansion of CAR-T cells would be controlled at the tumor sites without apoptosis. This is essential for avoiding CRS side effects caused by the over-activation of CAR-T cells, which could potentially lead to chronic inflammation or allergic or autoimmune disorders.

#### 2.1.1 Liposomal

Liposomes, an ideal drug-loading cargo widely applied in chemotherapy, possess advantages such as low immunogenicity, controlled drug release, and ease of chemical modification. Liposomes are typically anchored onto the external surface of CAR-T cells through a click reaction (Hao et al., 2020). The preparation process involved two steps. First, two-tailed lipids containing a hydrophobic fragment, such as 1,2-distearoyl-sn-glycero-3-phosphoethanolamine (DSPE) or cholesterol, were inserted into the lipid bilayers of CAR-T cell membranes through hydrophobic interactions. These lipids included a hydrophilic linker, such as polyethylene glycol5000 (PEG5000), with a tetrazine terminal for attaching the fabricated liposome structure. Second, liposomes were constructed using lipids derived from bicyclononyne (BCN), with DSPE-BCN being a suitable choice for the prepared lipids. Immunotherapy factors, such as avasimibe (an inhibitor that targets cholesterol esterification enzyme acetyl-CoA acetyltransferase І, elevating excess plasma membrane cholesterol and stimulating TCR signals to enhance T cell activity), IL-15, IL-21, and so on, could be loaded into the liposomes. Superagonist, a photosensitizer ICG, could also be loaded into the liposome. Subsequently, the DSPE-BCN liposome formulation loaded with the desired drug was subjected to a mild reaction with DSPE-PEG5k-Tre-anchored CAR-T cells for a duration of 0.5 h. The rapid click response ensured the integrity of CAR-T cell functions and the stability of NPs.

#### 2.1.2 Protein nanoparticles

Proteins, such as IL-15, IL-12, and antibodies, could be cross-linked by disulfide-based bis-N-hydroxy succinimide cross-linker (NHS-SS-NHS) for the formation of protein nanogels (Tang et al., 2018). The reaction should require an excess molar ratio of cross-linker to protein (for example, 15:1 cross-linker: IL-15). However, direct attachment of IL-15 or IL-12 nanogels onto CAR-T cells would result in a rapid internalization of these nanogels. To sustain stimulation, nanogel backpacks must be anchored onto T cell surfaces by monoclonal antibodies, such as antibody-CD11α and antibody-CD45, etc. By further cross-linking IL-15 nanogels with antibody-CD45, the IL-15 nanogels showed increased half-life on the T cells’ surfaces. A small portion of poly (ethylene glycol)-poly (L-lysine) (PEG-PLL) was also grafted onto the nanogels through another cross-linker, NHS groups. PEG-PLL provided the nanogels with a positive zeta potential, facilitating their initial electrostatic interaction between IL-15 nanogels and T cell plasma membranes, thereby offering a high efficiency of nanogel loading per T cell. IL-15 nanogels modified with anti-CD45 were maintained on raw, unstimulated T cells for at least 7 days. Once the T cells were activated by the attachment of tumor cells, the upregulated cell surface reduction caused the cleavage of the disulfide bond in the cross-linker (NHS-SS-NHS). IL-15 was then accelerated released to stimulate the expansion of CAR-T cells at the tumor sites. This approach helped mitigate the side effects of CRS by minimizing the off-target activation or proliferation of T cells.

Human serum albumin (HSA) has achieved safety and effectiveness in constructing nanomedicine for chemotherapy and is also an ideal nanbackpack material for CAR-T cells (Luo et al., 2022). To construct HSA NPs, HSA was reduced by adopting a certain amount of GSH at room temperature. Following the removal of excess GSH, a solution of reduced HSA at pH 7.5–8.0 was mixed with the superagonist IL-12. During this mixing process, the disulfide bonds were reconstructed. Subsequently, ethanol was added to precipitate IL-12-loaded HSA NPs, resulting in the formation of disulfide-bonded IL-12-SS-HSA NPs. IL-12-SS-HSA NPs were subsequently modified with dibenzocyclooctyl (DBCO) groups using DBCO-NHS. Then, DBCO-IL-12-SS-HSA was reacted with azide-sugar-derived CAR-T cells to achieve IL-12-SS-HSA-CAR-T. IL-12 could be quickly released by the breakage of disulfide bonds under conditions of CAR-T cell activation.

### 2.2 Construction of nanoparticles for improving TME

Therapeutic factors could be targeted delivered to the tumor site by NPs, remodeling the TME through radiotherapy and photodynamic and immunoregulation therapy (Figure 2). Different from NPs, CAR-T cells could function independently. Therefore, NPs should possess good drug-carrying and long-circulating capabilities *in vivo*.

#### 2.2.1 Polyester nanoparticles

Polyester polymers, such as polylactic acid (PLA), polycaprolactone (PCL), and poly (lactic-co-glycolic) acid (PLGA), which have been approved by the FDA as pharmaceutical excipients, could encapsulate photosensitizers such as indocyanine green (ICG) through a Pickering emulsion method (oil-in-water (o/w)) (Chen et al., 2019a). However, single ICG-PLGA nanoparticles could only be injected intratumorally due to their low water solubility. Therefore, hydroxyethyl starch was grafted onto the polyester polymer to create an amphipathic polymer, HES-PCL. The photosensitizer ICG and the immunosuppressor transforming growth factor-β (TGF-β) inhibitor LY could be co-loaded into the hydrophobic pores by the o/w Pickering emulsion method (Tang et al., 2023). The resulting ICG/LY@HES-PCL nanoparticle was targeted to the site of lymphoma through enhanced penetrability and retention (EPR) effects *in vivo*.

#### 2.2.2 Nanozymes

Nanozymes possess activities similar to natural enzymes and have often been used to regulate the tumor microenvironment (TME) through nanocatalytic chemical reactions with minimal adverse side effects *in vivo* (Liu et al., 2019; Yang et al., 2019; Xu et al., 2020; Zhu et al., 2020). The initial Fe-based nanozyme, which converted endogenous hydrogen peroxide (H_2_O_2_) to hydroxyl radicals (^.^OH), a highly toxic reactive oxygen species (ROS), could damage intracellular biomolecule substances (proteins, DNA, etc.) and directly killed cancer cells or trigger programmed cell apoptosis. Copper (Cu), with a wide range of accessible oxidation states and a high near-infrared (NIR) absorption rate (Zhu et al., 2021), was frequently used to construct nanozymes. The HA@Cu2-XS (HCNs) nanozyme could be prepared using the sacrificial template chemical transformation method. Briefly, CuCl_2_ was reacted with Na_2_S using HA as a capping stabilizer. This process involved the conjugation of Cu ions with HA carboxyl groups. HA could facilitate the assembly of the HA@Cu_2_-XS nanozyme and provided its stability *in vivo*.

### 2.3 Implantable biomaterials

**FIGURE 3:**
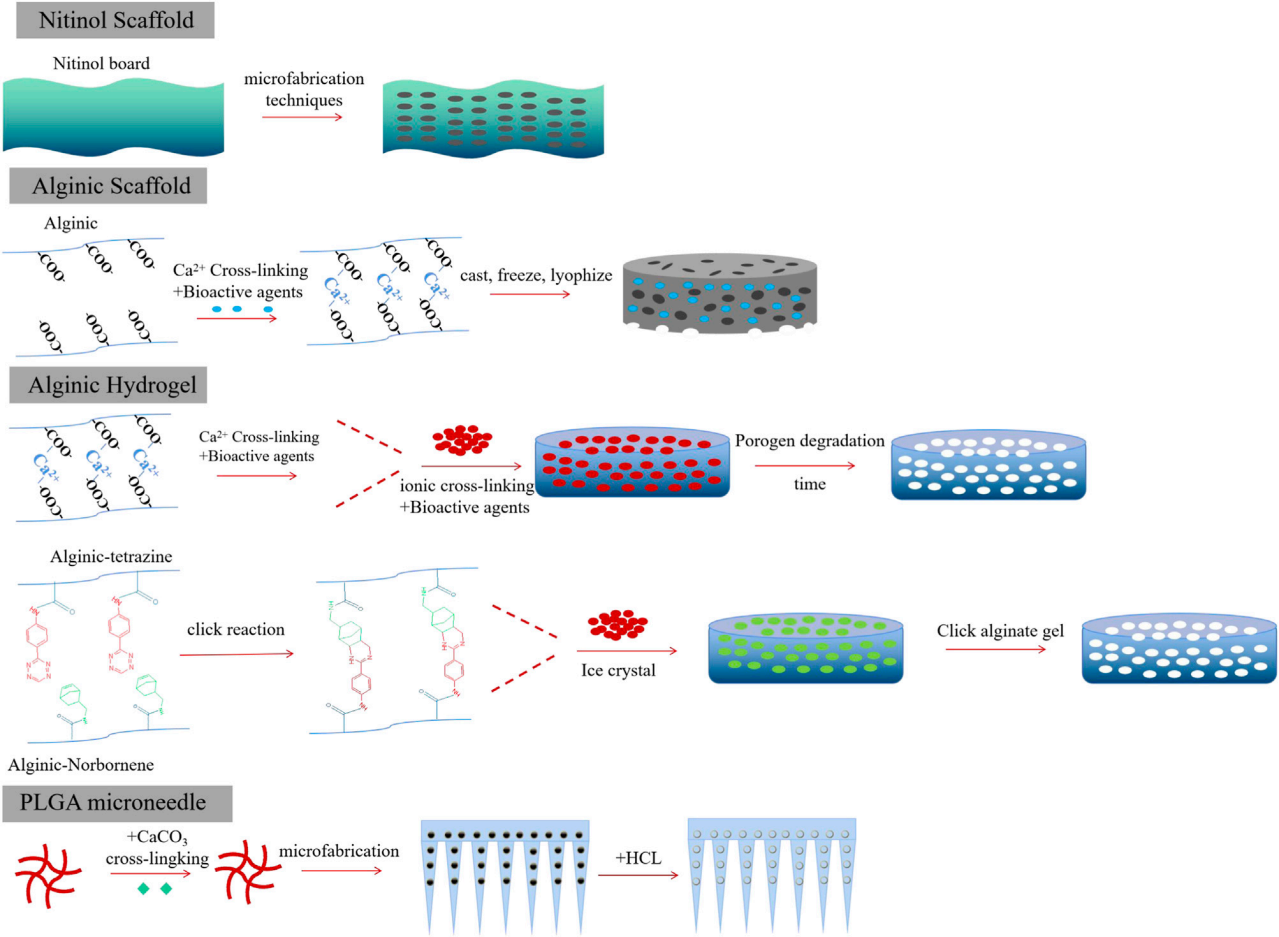
Different forms of implantable biomaterials facilitate CAR-T therapy toward solid tumors.

In general, implantable biomaterials should possess desirable porosities for the controlled release of therapeutic cargo. The materials should be inherently non-immunogenic, such as alginate, chitosan, and PLGA. Other methods for enhancing biocompatibility include adjusting the surface charge and topography (Bridges and García, 2008; Wen et al., 2016). Additionally, a key goal was to achieve scaffold biodegradation, which eliminates the need for surgical removal after therapy. Biodegradation could respond to enzymatic, hydrolytic, and pH stimuli and could undergo surface or bulk degradation. Biomaterials could be transported to tumor sites either through surgical implantation or by orthotopic injection. However, the delivery pathway was determined by the tumor site’s position and the physico-chemical properties of the biomaterials. The utility of designed biomaterial scaffolds also depended on their pore size and porosities (Kearney and Mooney, 2013). For example, macroporous scaffolds were commonly used to facilitate cell trafficking, while nanoporous scaffolds were specifically engineered for the targeted delivery of therapeutic agents to desired tissues. Importantly, the release kinetics of bioactive agents released from scaffolds is crucial in regulating the resulting immune response and the function of CAR-T cells (Bencherif et al., 2013). Release kinetics could be influenced by diffusion or environmental stimuli. Bioactive agents could be loaded into the scaffolds through covalent binding or van der Waals force, thus controlling their release kinetics to enhance CAR-T function while avoiding the CRS effect (Kearney and Mooney, 2013) (Figure 3).

#### 2.3.1 Porous alginate hydrogels and scaffolds

Alginate, a natural polysaccharide composed of β-D-mannuronic (M) and α-L-guluronic (G) sugar residues, possessed advantages such as low immunogenicity, controlled drug release kinetics, and desired degradation rates (Hwang et al., 2010; Lee and Mooney, 2012; Andersen et al., 2015). Due to these advantages, alginate had been approved by the FDA and widely used as an implantable biomaterial. The mechanical properties of gels based on alginate could be influenced by their molecular weight distribution, type, and extent of crosslinking (Ding et al., 2017). Alginate hydrogels could be designed to load therapeutic drugs, cytokines, and viral vectors for sustained release (Kuo and Ma, 2001; Lee and Mooney, 2012; Nayak et al., 2020). Additionally, the carboxylic acid groups on the alginate backbone could be easily chemically modified through the conjugation of polymers, drug conjugates, or proteins. Alginates could be cross-linked by divalent cations such as calcium or through a click cross-linking reaction. Compared to calcium cross-linked alginate hydrogels, click cross-linked hydrogels were safer and more retained in objective tissues, receiving only minor inflammatory host responses (Christopher et al., 2020; Agarwalla et al., 2022). They could be locally fixed at the injection site and facilitated the capture of small molecules from the circulation. Pore-forming alginate gels could be fabricated by adding degrading alginate porogens. The degradation process of alginate gels was managed by the oxidation and reduction ratio (Verbeke and Mooney, 2015), thus facilitating the formation of pores within the alginate gels.

Porous alginate scaffolds could also be fabricated using a cryogelation method (Madelyn et al., 2022). First, alginate was dissolved in molecular biology-grade water and then mixed with CAR-T stimulating antibodies, such as anti-CD3 and anti-CD28, at 4°C overnight. Afterward, alginate and recombinant human cytokines, such as IL-15 and IL-2, were added, and all the agents in the solution were stirred for 15 min. Finally, a certain amount of calcium gluconate was added, and the mixture was vigorously stirred to achieve cross-linked alginate. The resulting mixture was then frozen at −20°C for 24 h and lyophilized. Porous alginate scaffolds should be stored at 4°C prior to further use *in vivo*.

#### 2.3.2 Short synthetic DNA scaffolds

DNA scaffolds were developed using PLGA polymer-based micrometer-sized immune cell-engaging particles (ICEp) (Xiao et al., 2021). PLGA was cross-linked with PVA to create PLGA particles of varying sizes. DNA was thiol modified to produce DNA oligonucleotides polymer. Then, payload-attachable DNA scaffolds with continuously increasing density were formed by mixing PLGA10k-PEG5k-maleimide and thiol-modified DNA with varying ratios using the emulsion method. This method allowed for a high level of surface particle loading density (roughly 5 million DNA molecules per NP). By utilizing hybridization-guided particle loading, it achieved approximately 27-fold higher efficiency compared to the traditional methods (Schmid et al., 2017). Intriguingly, multiple cargos could be controlled loaded onto one material using single DNA nucleotide sequences. By preparing DNA-scaffolded particles with different diameters and loading bioactive molecules, their applications could be explored, ranging from intracellular cargo delivery to extracellular signal transduction.

### 2.4 Porous microneedle patch

However, tumor recurrence could be delayed by removing tumors through surgical intervention, which exposes the remaining tumor cells to endogenous T cells (Brown et al., 2016; Xu et al., 2018). These residual tumor fragments seriously hinder the tumor-targeted delivery of CAR-T cells (Chen et al., 2019b). Therefore, developing a polymeric porous microneedle (PMN) patch to load CAR-T cells for precisely delivering these cells to the surgically removed tumor site is of great significance (Li et al., 2021). A porous microneedle patch, crafted from a porous material with numerous small holes, serves as an effective tool for CAR-T delivery and therapy. Thus, the biocompatible PLGA and CaCO_3_ microparticles (MPs), with a diameter of 8 μm, were mixed and then posted into a polydimethylsiloxane (PDMS) micromold. PLGA was cross-linked by dioxane pre-injected into the needle at 90 °C overnight, and then, the microneedle patch was removed or detached. The microneedle patch was soaked in a hexane/HCl solution and swelled for 2 h, after which water was added to stop the reaction between CaCO_3_ and HCl, coupled with the generation of CO_2_ bubbles. Finally, a hydrophilic surface was generated on the PMN patch by treating it with plasma. Subsequently, PLGA PMN was mixed with CAR-T solution and treated with a vacuum, CO_2_ bubbles were removed, and CAR-T cells were captured by the porous PMN. The PLGA microneedle patch offered a high-capacity and precise delivery tool for CAR-T cells to target and kill tumors by overcoming the physical obstacles in solid tumors.

## 3 Biomaterials for overcoming three obstacles

### 3.1 Trafficking and infiltration obstacles

The presence of an abnormal vascular microenvironment and cross-linked matrix barrier was the primary reason that hindered CAR-T cell recruitment. The infiltration of T cells to tumor sites underwent a transformation process (Yong et al., 2017). Upon stimulation by soluble chemokines, the surface microvilli of T cells were rapidly disintegrated. The cortical actin skeleton released integrins, which increase T cell activity. This process encouraged T cells to adhere to endothelial cells and migrate across blood vessels to tissues. T cells in the tissue further formed a trailing tail foot, which projected on the surface of the cell matrix, producing a high traction force and promoting the cells’ movement toward tumor tissues (Burkhardt et al., 2008). Tumor vascular spaces usually varied from 0.2 to 1 μm, much smaller than the diameter of T cells (5–7 μm). Therefore, even if CAR-T cells recognized the tumor antigen, it was a long journey for their migration to the tumor sites. To address these challenges, the conventional approach is to directly inject CAR-T cells into tumor tissues, which has been proven effective in promoting cell trafficking (Van et al., 2013). In clinical settings, intratumoral delivery of CAR-T has been employed for treating various types of cancers, including glioblastoma, mesothelioma, adenocarcinoma, and breast, among others (Beatty et al., 2014; Katz et al., 2015; Brown et al., 2016; Tchou et al., 2017). Although these studies had shown the feasibility and safety of CAR-T cell administration, the limited durability of post-inoculated CAR-T cells had restricted their benefits in clinical settings. Therefore, it is urgent to develop more feasible clinical CAR-T transporting technologies.

One strategy was to directly administer CAR-T cells to lesion sites through an implanted scaffold system. M. E. Coon et al. employed nitinol, an inert metal widely used for healthcare devices, to fabricate a nitinol mesh film with a periodic porous structure through microfabrication techniques (Coon et al., 2020). The nitinol mesh, with a pore width of 22.4 µm and a length of 146.3 µm, was seeded with cells onto a fibrin matrix. The nitinol mesh was further modified with antibodies to enhance the functions of CAR-T cells. An ovarian tumor model was established in mice, and nitinol-delivered CAR-T cells were implanted into the tumors. While intratumoral or intravenous injection of CAR-T cells only demonstrated temporary therapeutic effects, the nitinol-based scaffold group showed significant eradication effects. The nitinol-based scaffold could also prevent tumor invasion. Results from a tumor invasion assay *in vitro* using a collagen gel embedded with the stents and tumor cells were showcased. In the mesh group blended with CAR-T cells, tumor size decreased to 29.7%, whereas the group without the treatment of CAR-T cells exhibited over 99% tumor volume in the tissues. Thus, the nitinol-based scaffold provided a tumor-targeted CAR-T cell delivery system, leading to enhanced antitumor efficacies and a low tumor metastasis rate. However, considering that the nitinol-based scaffold should be removed after the therapy, it might not be practical in a clinical setting.

**FIGURE 4:**
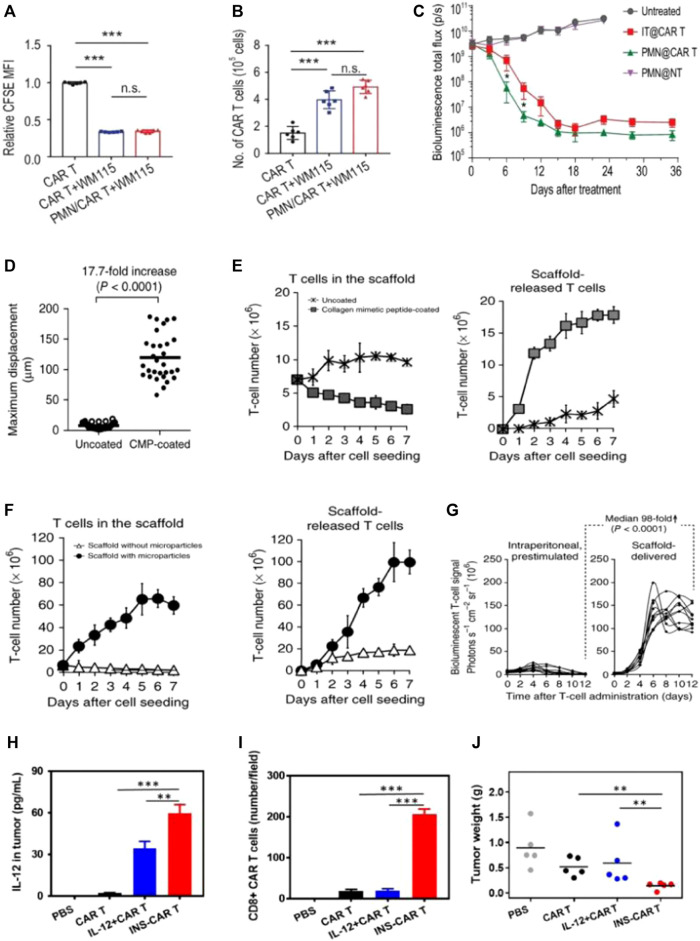
Biomaterial-based delivery of CAR-T cells to overcome trafficking or infiltration obstacles. **(A–C)** PMN patches for CAR-T cells’ traffic and infiltration. Adapted with permission (Li et al., 2021). **(A,B)** Patches enhance CAR-T infiltration *in vitro*; **(C)** Delivering CAR-T cells via exhibited patches improved antitumor activities in pancreatic tumors (kinetics). **(D–G)** Biopolymer implants improve adoptive therapy efficacy. Adapted with permission (Stephan et al., 2015). Porous polysaccharide scaffolds facilitate rapid migration **(D)**, substantial expansion **(E)**, and prolonged release **(F)** of T-cells; **(G)** CBR-luc signal intensities following T-cell transfer indicate significant expansion of T cells with tissues. **(H,I)** IL-12-loaded nanobackpacks remarkedly improved INS-CAR-T therapy effectiveness in combating solid tumors. Adapted with permission (Luo et al., 2022). **(H,I)** The INS delivery system induced CAR-T cells’ infiltration to orientated sites; **(J)** The INS-CAR-T immunotherapeutic effects against tumors *in vivo*.

Biodegradable multifunctional scaffolds have been further developed. Hydrogels were water-swollen three-dimensional networks of hydrophilic polymers, cross-linked to form structures that had found extensive applications across various fields for the past 5 decades. Tsao’s group developed a thermal, biocompatible, biodegradable, and low-immunogenicity hydrogel known as poly (ethylene glycol)-g-chitosan (PCgel) hydrogel. This hydrogel was developed as a reservoir to load and release CAR-T cells for immunotherapy targeting brain tumors (Tsao et al., 2014). PCgel was liquid at low operating temperatures and underwent gelation at healthy body temperatures, enabling a sustained release of Anti-EGFR CAR-T cells to brain tumors without the need for surgical intervention. PCgel facilitated Anti-EGFR CAR-T to achieve increased anti-glioblastoma activity as compared with Matrigel. A thermogel formulated by chitosan and NaHCO3 in the buffer exhibited excellent mechanical qualities and biocompatibility, rendering it a desirable option for CAR-T cells’ local delivery via catheter or needle. Abigail K. Grosskop’s group developed a transient injectable hydrogel (PNP hydrogels) with stimulatory properties by dodecyl-grafted hydroxypropyl methylcellulose (HPMC-C12) and arginine–glycine–aspartic acid (RGD)-decorated PEG-PLA NPs (Anisha et al., 2022). These two types of materials resulted in the formation of a robust hydrogel, facilitated by entropy-based associations and dynamic doublication. CAR-T cells could be attached to RGD, and their motility and viability could also be enhanced. IL-15 (Mw = 15 kDa), with a short elimination half-life (t1/2 = 1.5 h) *in vivo*, was easily encapsulated within the hydrogels through simple mixing during the fabrication process. IL-15 could be non-specifically adhered to the hydrogel mesh, and it showed a superior sustained IL-15 release effect. It was demonstrated that the majority of the encapsulated IL-15 remained (>80%) within these gels even after 7 days *in vivo*, where there was an excess of surrounding medium to prevent loss by diffusion. PNP hydrogels could entrap CAR-T cells and enable their viability, prolonged retention, and sustained activation. The PNP-CAR-T hydrogel was then safely and easily subcutaneously injected, producing potent distal antitumor responses, which were crucial for expanding the applicability of these treatments. Zongchao Han et al. developed injectable chitosan-PEG hydrogel@GD2.CAR-T to deracinate retinoblastoma (RB) in a preclinical model. GD2 served as a neuroepithelial tumor marker (Wang et al., 2020). As a control, CD19 CAR-T cells (10^–6^ cells) were administered either through intratumoral injection or by means of injection into subretinal membranes. CAR-Ts with GD2 showed limited efficacy in controlling retinoblastoma (RB) growth, with all mice eventually developing tumors and even requiring euthanasia at the end of 70 days. By incorporating IL-15 and encapsulating it with injectable hydrogels prepared by chitosan-PEG, the lifespan and biodistribution of GD2-targeted CAR-T cells were improved. The use of injectable hydrogel enabled localized delivery of T cells, reducing inflammation and retinal detachment. Therefore, injectable hydrogel facilitated GD2. CAR-Ts to achieve superior anti-tumor effects. GD2. CAR-Ts effectively eradicated RB tumor cells without causing any detrimental effects on mouse vision. Stephan’s team created a scaffold with a microporous structure using alginate polymers, which incorporated a peptide with similar collagen properties for T cell binding and silica MPs within the scaffold’s void spaces (Stephan et al., 2015) (Figures 4D–G).

Hydrogel materials also provided an ideal survival environment for CAR-T viability. Gu’s team devised a biocompatible microneedle patch made of PLGA with a porous structure. The patch with CAR-T cells was implanted into tumors following tumor removal, aiming to impede tumor growth and prevent recurrence (Li et al., 2021). The presence of pores at the tips of the microneedles facilitated the uniform dispersion of loaded CAR-T cells, ensuring their bioactivity remained intact without any loss (Figure 4B). As compared with intratumoral injection, the exhibited microneedle delivery system enhanced the infiltration and immune stimulation of CAR-T cells. This approach significantly suppressed tumor recurrence and inhibited tumor growth in postoperative resected melanoma or orthotopic pancreatic tumors (Figure 4C).

Hu’s team designed NPs (LY/ICG@HES-PCL) based on hydroxyethyl starch-poly (ε-caprolactone) (HES-PCL), which were loaded with LY2157299 (LY, a TGF-β inhibitor) and the photosensitizer indocyanine green (ICG) (Tang et al., 2023). The LY/ICG@HES-PCL upregulated chemokines CXCL9/10/11, which were involved in the migration of CAR-T cells to specific sites. This upregulation facilitated CAR-T cells’ accumulation at lymphoma sites. Additionally, combining CAR-T cell treatment with LY/ICG@HES-PCL also accelerated cell differentiation, resulting in a 2.4-fold enhancement of the antitumor activity of CAR-T cells, surpassing the efficacy achieved with CAR-T cell treatment as a standalone approach. Cai’s work developed human serum albumin-based NPs to load interleukin-12 (IL-12) and CAR-T cells, which were called INS-CAR-T (Luo et al., 2022). It revealed that INS-CAR-T cells, when blended with 3D tumor spheroids, resulted in a remarkable increase of more than 2.8 times in antitumor ability as compared with the use of INS-CAR-T cells alone. Moreover, INS-CAR-T cells exhibited the ability to release IL-12 upon encountering elevated glutathione levels within the microenvironments of tumors (Figure 4H). The results revealed that the use of IL-12-loaded nanobackpacks markedly improved CAR-T cells’ therapeutic effectiveness for combating tumors (Figure 4J).

### 3.2 Activation obstacles

Solid tumors characterized by slight acidity, hypoxia, and nutritional deficiency can cause limited effects after treatment with CAR-T cells. Several tactics utilizing biomaterials have been investigated that can modify the hostile microenvironment of solid tumors. These approaches include stimulating the production of inflammatory cytokines, reprogramming immune regulatory cells, and inhibiting immune checkpoints.

**FIGURE 5:**
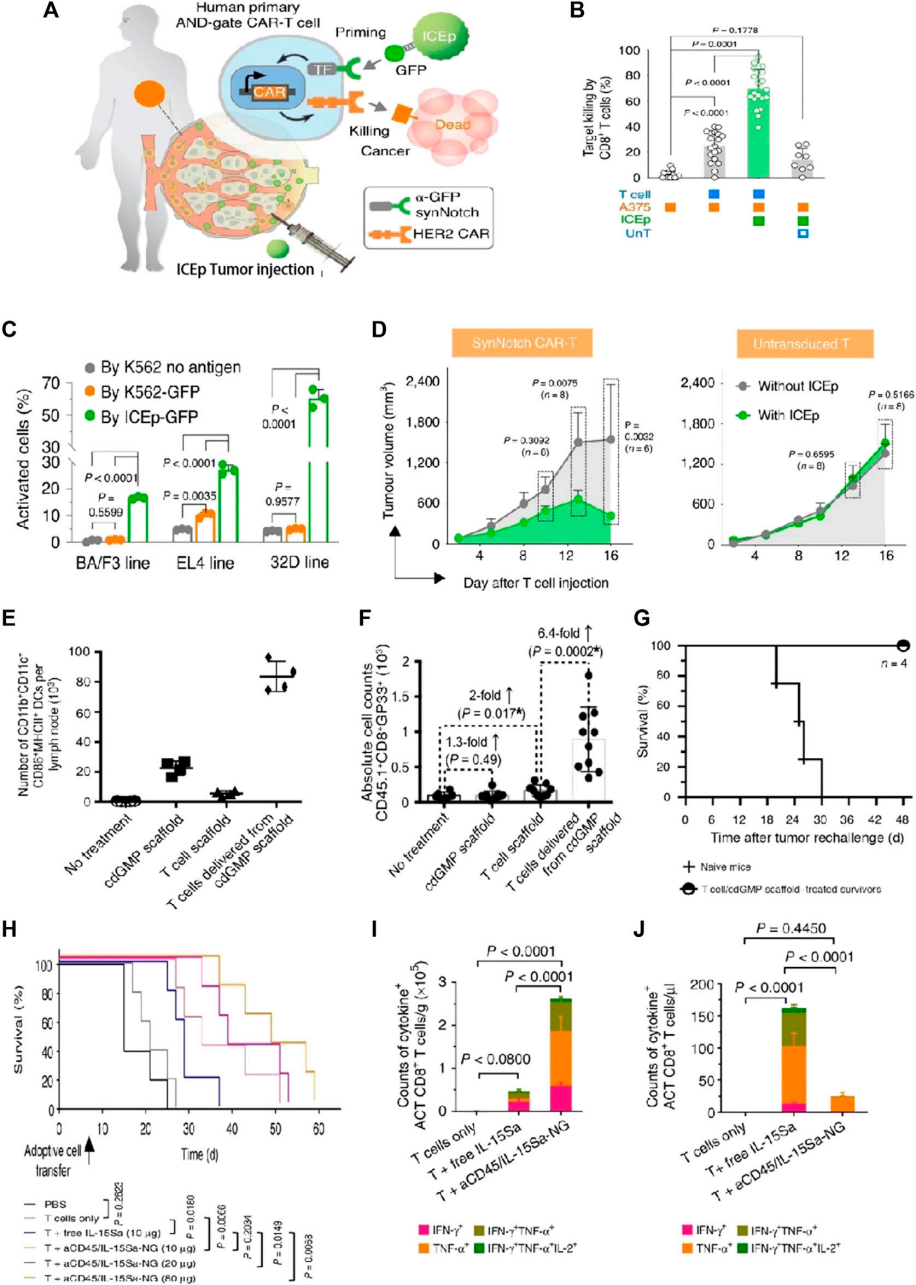
Biomaterial-based delivery for CAR-T cells to overcome activation obstacles. **(A–D)** DNA scaffolds enhanced the CAR-T cells’ localized activation, which was equipped with an AND-gate system. Adapted with permission (Xiao et al., 2021). **(A)** Schematic diagram; **(B)** Stimulation of CD8^+^ AND-gate CAR-T cells *in vitro*; **(C)** The BFP^+^ groups of murine immune cells were stimulated by ICEp-GFP; **(D)** The sizes of the tumor and the tumor on the opposite side, measurements after treatment with CAR-T cells, and untransduced T cells; **(E–G)** Scaffold-based CAR-T cells with STING activate host APCs to eliminate heterogeneous tumors. Adapted with permission (Smith et al., 2017). **(E)** The total counts of mature and activated DCs in peripancreatic lymph nodes; **(F)** The simultaneous release of cdGMP and CAR-T cells effectively sensitizes lymphocytes with tumor-specific reactivity; **(G)** Implants elicit global antitumor immunity. **(H–J)** IL-15Sa-NGs backpacks enhance T cell therapy through promoting selectively transferred T cell proliferation adoptively within tumor tissues. Adapted with permission (Tang et al., 2018). **(H)** Survival curves of Thy1.2^+^ C57Bl/6 mice through different therapies; **(I)** Quantification of polyfunctional CD8^+^ T cells from adoptive cell transfer in tumors; **(J)** The utilization of IL-15Sa-NG extends optimal treatment window for delivering cytokines during ACT.

Although anti-CD19 CAR-T cells have shown significant success, the main challenge encountered is the loss of CD19 antigen at the tumor site, which leads to CD19 antigen-negative relapse. Smith et al. introduced implantable biopolymer scaffolds that allowed for the co-administration of interferon gene agonists (named STING) with CAR-T cells (Figures 5E–G) (Smith et al., 2017). Cyclic di-GMP (cdGMP) could activate pathways of STING in antigen-presenting cells (APCs), thereby efficiently triggering responses for endogenous immune. To recruit APCs while priming T cells, STING was encapsulated within mesoporous silica MPs incorporated into the biopolymer scaffolds. In a murine model bearing tumors, the release of cdGMP in tumors resulted in a markedly greater quantity of specifically primed T cells within the scaffolds co-delivering cdGMP, with an approximately 6.4-fold increase compared to scaffolds containing T cells only. Thus, mesoporous silica MPs merged with the biopolymer scaffold STING agonist, leading to the establishment of a linked immune response toward antigen-negative tumor cells, even in the absence of CAR-T cells.

Immune checkpoints at solid tumor sites presented another physiological barrier to the activities of CAR-T cells. It has been demonstrated that multiple receptors on T cells could trigger inhibitory signals, including cytotoxic T-lymphocyte-associated protein 4 (CTLA-4), T cell immunoglobulin domain and mucin domain-3 (TIM3), and programmed cell death protein 1 (PD-1). Consequently, hydrogel-based biomaterials have also been employed in the modulation of immune checkpoints. To prevent postoperative tumor recurrence, Gu’s group successfully integrated CAR-T cells, IL-15@PLGA NPs, and platelets decorated with anti-PD-L1 antibodies within a biodegradable hyaluronic acid hydrogel (Hu et al., 2021). Following tumor resection, the inflammatory microenvironments at the tumor sites could stimulate platelets to generate platelet-derived particles (PMPs), which released anti-PD-L1 antibodies. Simultaneously, sustained release of IL-15 from PLGA NPs occurred within the same microenvironments. Anti-PD-L1, together with IL-15, could trigger the activation and proliferation of CAR-T cells. Compared to injecting intratumorally or intravenously, the platform exhibited increased activation of CAR-T cells in the operative areas and achieved superior tumor inhibition rates in a murine melanoma model. Furthermore, the CAR-T hydrogel system also demonstrated effective inhibition of tumor progression in distal locations.

Huang’s team introduced a biocompatible approach called immune cell-engaging particles (ICEp), utilizing scaffolds based on chemo-synthetic short DNA to enable efficient and adjustable protein loading (Coon et al., 2020). These particles allowed for the loading of a series of immunomodulators, including antigens, checkpoint inhibitors, co-stimulatory ligands, and cytokines, while maintaining their bioactivity. Notably, the antigen-presenting ICEp exhibited the ability to locally control CAR-T cell (AND-gate) activation, leading to effective tumor elimination *in vivo*. Furthermore, IL-2 was loaded onto ICEp surfaces, influencing T-cell activation and expansion. The integrated system held promise for enhancing immunotherapies by enabling precise immune modulation at the tumor sites. Utilizing DNA-based scaffolds as substrates offered distinct advantages for a broad range of modulatory biomolecules. These scaffolds could effectively load checkpoint inhibitors, adjuvants, accommodate cytokines, antigens, agonistic or antagonistic antibodies, and other molecules, allowing for precise regulation of the local environment and enhancing CAR-T therapy efficacy.

Hydrogel systems co-transporting oxygen could facilitate CAR-T cell activation. Luo’s work presented an injectable hydrogel-based platform incorporating an immunomodulatory microarray system for the targeted delivery of CAR-T cells (Luo et al., 2020). By intratumorally injecting CAR-T cells, the gel layer of the hydrogel surrounding and coating the microchips (MCs) rapidly degraded. This degradation facilitated the delivery of oxygen carriers known as HEMOXCell. HEMOXCell possessed exceptional oxygen storage capabilities, with each Hemo molecule capable of storing 156 oxygen molecules. The continuous release of oxygen within the tumor microenvironment promoted a high intratumoral oxygen tension, which created a favorable milieu for the survival and activity of infiltrating immunocytes. This sustained oxygen supply not only supported the effectiveness of CAR-T cell therapy but also had the potential to downregulate the expression of a protein associated with cancer progression, hypoxia-inducible factor-1α (HIF-1α). By modulating the tumor microenvironment, the controlled release of oxygen contributed to enhanced immunocyte function and inhibited factors that drive malignant tumor growth.

The therapeutic effectiveness of CAR-T cells could be enhanced through surface engineering with NPs loaded with therapeutic drugs. Kwong et al. devised a gene switch that responds to heat and integrated it into CAR-T cells, enabling targeted therapy for tumors in conjunction with the photothermal characteristics of gold nanorods (Ian et al., 2021). This delivery system provided a novel approach to control intratumoral CAR-T cell activity using synthetic gene switches responsive to mild temperature elevations (40°C–42°C). CAR T cells were photothermally activated through gold nanorods, leading to transgene expression exclusively within the tumor sites on the mouse model. Additionally, the system demonstrated heightened antitumor efficacy and successfully countered antigen escape in murine models of adoptive transfer. Tang et al. presented an innovative method to load substantial protein drugs onto T cells through the use of nanogels (NGs) that precisely released agents upon activation of the T cell receptor (Tang et al., 2018). The results showed that the “backpack” T cells selectively released the IL-15 super-agonist complex upon recognition of tumor cells (Figure 5I). T cells within tumors exhibited a 16-fold specific expansion when employing NGs compared to the systemic administration (Figure 5J). Furthermore, NGs led to 80% complete tumor eradication in mice on the xenograft mouse model, highlighting their therapeutic efficacy (Figure 5H).

### 3.3 Proliferation and survival obstacle

Gelatin-based hydrogels have the advantages of being biocompatible, biodegradable, and possessing desirable porosities, providing a desired survival environment for CAR-T cells. In the study conducted by Suraiya’s group, a biocompatible gelatin-based microgel system was developed as a three-dimensional (3D) culture platform to support CAR-T cells’ survival. Researchers implemented a microfluidic device incorporating a pipette tip, facilitated by a biorthogonal photo-click reaction, to enable the crosslinking of gelatin modified with norbornene together with PEG decorated with thiol groups (Tsao et al., 2014). The CAR-T cells encapsulated within gelatin-based microfluidic microgels exhibited excellent bio-viability (>87%) even after 1 week, demonstrating comparable cell growth levels under standard cultivation circumstances.

The CAR structures, starting CAR-T population, culture techniques, co-stimulatory factors, and final CAR-T composition were key contributors to CAR-T cell proliferation and persistence *in vivo*. To induce CAR-T proliferation, the most commonly employed methods include the use of anti-CD3/CD28 dynabeads or antibodies (Chang et al., 2007). Agarwalla’s study devised a groundbreaking approach by creating an implantable multifunctional alginate scaffold, known as MASTER, for CAR-T cell engineering and release. This innovative scaffold enabled CAR-T cells’ rapid manufacturing *in vivo* and dramatically shortened the production time frame to just 1 day (Pritha et al., 2022). Anti-CD3 and CD28 antibodies were modified onto MASTER (serving as an appropriate interface for gene transfer, mediated by viral vectors), while human peripheral blood mononuclear cells and retroviral particles encoding CD19 were seeded. Upon implantation subcutaneously, it facilitated CAR-T cells’ well-organized release in a mice model. The generated CAR-T cells *in vivo* could enter the bloodstream and then effectively control the growth of distant tumors in murine lymphoma (xenograft model), thus exhibiting superior durability as compared with original CAR-T cells.

**FIGURE 6:**
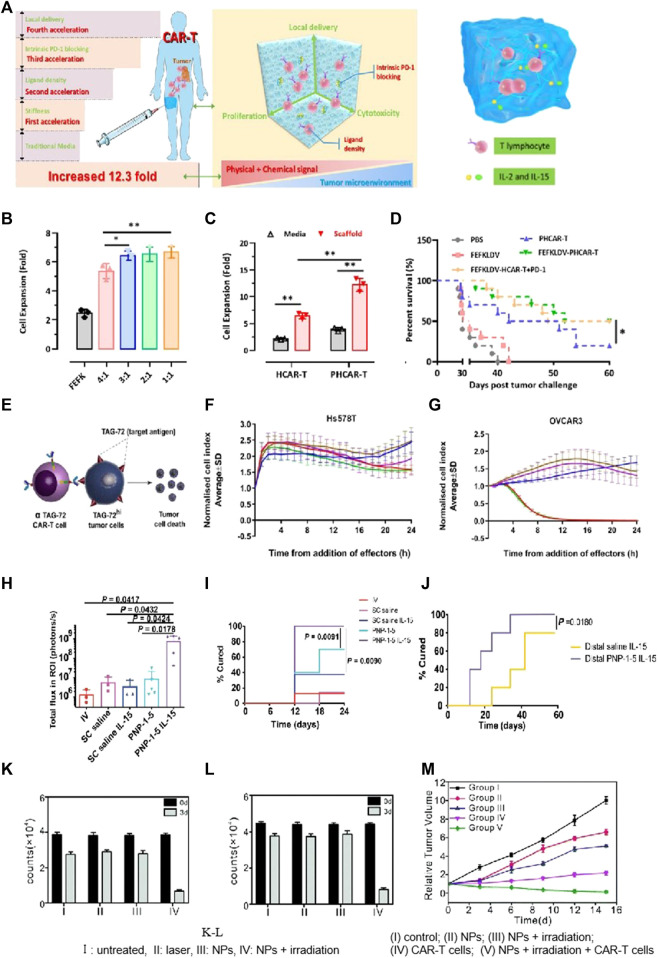
Biomaterial based delivery for CAR-T cells to overcome proliferation and survival obstacles. Tailored peptide-based hydrogel scaffolds for promoting CAR-T cell proliferation and enhancing tumor immunotherapy. Adapted with permission (Jing et al., 2022). **(A)** Illustrative diagram; **(B)** The scaffold matrix promoted cell proliferation; **(C)** PD-1 scFv in the scaffold enhanced HCAR-T cell proliferation; **(D)** Scaffold encapsulation enhances PHCAR-T anti-tumor effect. **(E–G)** αTAG-72-CAR-T cells specifically recognize TAG-72 antigens and initiate targeted murder of the tumor cells. Adapted with permission (Abigail et al., 2022). **(E)** Schematic diagram; **(F–G)** Cytotoxicity of αTAG-72 CAR-T cells. **(H–J)** Polymer-NP (PNP) hydrogels improve T cells’ expansion and treatment efficacy of solid tumors. Adapted with permission (Anisha et al., 2022). **(H)** T cell signaling compared across groups on day 21; **(I)** PNP hydrogels improve antitumor efficacy; **(J)** PNP hydrogels effectively treat subcutaneous medulloblastoma in mice. **(K–M)** FA-Gd-GERTs NPs blended with CAR-T cells for excellent NHL therapy. Adapted with permission (Luo et al., 2022). CAR-T cells under the different conditions of JEKO **(K)** or RAJI **(L)**, indicating the NPs promote CAR-T cell proliferation after photothermal therapy.

The hydrogel structure and its components were critical for CAR-T cell proliferation. It has been reported that loosely cross-linked hydrogels offer highly expandable spaces for cellular division. Jie’s group created scaffolds for immune cell loading using peptide-based self-assembling hydrogels. This novel approach aimed to preserve and enhance CAR-T cells’ phenotypes (Jing et al., 2022). This self-assembled peptide consisted of hydrophilic and hydrophobic amino acids, resulting in the formation of two distinct surfaces. Additionally, the peptide exhibited a balance of disparate charges, which facilitated improved β-sheet conformation. The peptide hydrogels at the nanoscale acted as promising scaffolds for CAR-T immobilization and activation (Figures 6B,C). Hydrogels with optimal adhesive properties and stiffness had the ability to regulate cell viability through mechanotransduction signals, thereby facilitating both CAR-T cells’ activation and their proliferation. The peptide hydrogel scaffold-loaded CAR-T was injected into the tumor site. The developed approach achieved a remarkable 12-fold rapid amplification of CAR-T cells within a span of 3 days, surpassing the efficiencies of traditional protocols. Moreover, the local CAR-T cell delivery via scaffolds resulted in prolonged retention, effectively suppressing tumor progression and promoting CAR-T cells’ infiltration, surpassing the outcomes observed with traditional CAR-T treatments.

IL-15, a super-agonist that stimulates CAR-T cell proliferation, is usually co-administered via injection with a scaffold (Hurton et al., 2016). Stephan’s work adopted a biopolymer scaffold based on alginate for delivering CAR-T cells. This scaffold was fabricated using polymerized alginate combined with collagen-mimetic peptides (Stephan et al., 2015). IL-15 was used as the soluble factor loaded into silica MPs. The membrane bilayer of silica MPs was coupled with anti-CD3/CD28/CD137 antibodies. The incorporation of MPs into the scaffold led to a remarkable increase (22.0 times) in CAR-T cell proliferation and an excellent enhancement (8.3 times) in migration into the adjacent collagen gel within a span of 7 days. To visualize the distribution of CAR-T cells at the site of resection, a resection model of 4T1 breast tumor was established. Released CAR-T cells exhibited effective infiltration into the residual tumor, resulting in the suppression of tumor growth for a remarkable duration of over 80 days. This resulted in a 98-fold increase compared to direct intra-peritoneal CAR-T cell infusion. For the experimental group receiving CAR-T-bearing scaffolds, no relapse was observed, while in the control groups, mortality was evident. This microparticle-modified alginate scaffold reported in the study effectively enhanced CAR-T cell proliferation, survival, and therapeutic efficacy. Appel’s team produced a polymer-NP hydrogel (PNP hydrogel) consisting of a polymer prepared by HPMC and IL-15-loaded NPs (Abigail et al., 2022). The mixture with CAR-T cells was loaded into the PNP hydrogel and subsequently injected in close proximity to murine tumors (Figures 6H, I). Upon exposure to IL-15, these CAR-T cells were activated and then underwent proliferation, leading to the effective destruction of tumor cells. Remarkably, within a period of 12 days, the tumors, including the distal tumor, were completely eradicated (Figure 6J).

Interleukin-13 receptor (IL-13Rα2), known to be selectively overexpressed in approximately 75% of tumors, was commonly employed as a target for CAR-T cells’ treatments. Dong et al. loaded doxorubicin (DOX) onto IL-13-targeted NPs and conjugated them to the surfaces of CAR-T cells for the chemotherapeutic management of glioblastoma (Gloria et al., 2020). Their study demonstrated that the modification of CAR-T cells’ surfaces using “backpack” NPs methods led to a higher DOX accumulation and an increased presence of CAR-T cells at the tumor site. Moreover, TQM-13 CAR-T-modified cells exhibited a notable increase in the uptake of BPLP-PLA NPs within tumors, surpassing the uptake achieved with injections of NPs alone.

NP-based delivery has been demonstrated to be valuable for achieving multifunctional diagnosis and treatment in CAR-T therapies. Ye et al. prepared ibrutinib-loaded multifunctional therapeutic drug NPs (FA-Gd-GERTs) combined with CD19. CAR-T cells for the multimodal imaging and therapy of non-Hodgkin lymphoma (NHL) (Luo et al., 2022). This study reflected that FA-Gd-GERTs exhibited higher and more rapid accumulation in tumors compared to non-targeted NPs, making them highly effective for multimodal tumor imaging (Figure 6K-L). Additionally, FA-Gd-GERTs@ibrutinib demonstrated excellent photothermal conversion efficiency, leading to physiological, physical, and chemical changes within the tumors’ milieu triggered by photothermal effects. This further promoted the CD19. CAR-T cells’ infiltration and accumulation to greater extents (Figure 6M).

Tailored peptide-based hydrogel scaffolds for promoting CAR-T cell proliferation and enhancing tumor immunotherapy. Adapted with permission. (Jing et al., 2022). (A) Illustrative diagram; (B) The scaffold matrix promotes cell proliferation; (C) PD-1 scFv in the scaffold enhances HCAR-T cell proliferation; (D) Scaffold encapsulation enhances PHCAR-T anti-tumor effect. (E-G) αTAG-72-CAR-T cells specifically recognize TAG-72 antigens and initiate targeted murder of the tumor cells. Adapted with permission. (Abigail et al., 2022) (E) Schematic diagram; (F-G) Cytotoxicity of αTAG-72 CAR-T cells. (H-J) Polymer-NP (PNP) hydrogels improve T cells’ expansion and treatment efficacy of solid tumors. Adapted with permission. (Anisha et al., 2022) (H) T cell signaling compared across groups on day 21; (I) PNP hydrogels improve antitumor efficacy; (J) PNP hydrogels effectively treat subcutaneous medulloblastoma in mice. (K-M) FA-Gd-GERTs NPs blended with CAR-T cells for excellent NHL therapy. Adapted with permission. (Luo et al., 2022). CAR-T cells under the different conditions of JEKO (K) or RAJI (L), indicating that NPs promote CAR-T cell proliferation after photothermal therapy. (I) Untreated, (II) laser, (III) NPs, (IV) NPs + irradiation; (M) Tumor growth curves after various treatments. (I) Control; (II) NPs; (III) NPs + irradiation; (IV) CAR-T cells; and (V) NPs + irradiation + CAR-T cells.

## 4 Challenges and outlook

CAR-engineered T cell therapy, a novel cancer immunotherapy approach for hematology, has become one of the mainstream treatment strategies in clinical practice. Biomaterials have expanded the scope of application to solid tumors. However, many challenges need to be overcome in the fabrication of biomaterials for perfecting CAR-T therapy. Firstly, the safety and efficacy of biomaterials in CAR-T should be considered due to their complex composition. The US FDA-approved materials for nanomedicine are characterized by their simplicity and reproducibility. However, most of the biomaterials applied in CAR-T treatments have a complex structure and introduce different types of bioactive or chemical ligands. This complexity poses significant difficulties in achieving high-quality, clinically scalable production. Therefore, at the beginning of designing biomaterials for CAR-T therapy *in vivo*, the feasibility of its clinical application should be taken into consideration, including production cost, stability, repeatability, and the technology process of scale production. From a quality control perspective, small molecule biomaterials facilitate industrial production more than polymer biomaterials. In addition, the safety of CAR-T cell therapy should also be considered, specifically the side effects of CRS. CRS, an increased secretion of pro-inflammatory cytokines caused by the overactivation of CAR-T cells, could be potentially avoided by controlled-release designation of biomaterials. One potential approach is to combine the therapy with an immunosuppressor such as dasatinib. Dasatinib could regulate the activation status of CAR-T cells by inhibiting tyrosine kinase. Once CRS occurs *in vivo*, biomaterials should be designed to be compatible with immunosuppressors to regulate the levels of activated CAR-T cells and inhibit CRS.

## 5 Conclusion

CAR-T cell therapy targeting solid tumors has faced multiple challenges and obstacles that need to be overcome in order to improve its clinical efficacy. These challenges include trafficking and infiltration, activation, proliferation, and survival obstacles. Trafficking and infiltration obstacles restrict the migration of CAR-T cells to tumor sites. Activation obstacles, resulting from tumor heterogeneity and the tumor microenvironment (TME), could negatively impact the functions of CAR-T cells. Furthermore, the acidic, low-nutrient, and hypoxic microenvironment results in limited CAR-T cell proliferation, activation, and survival properties. Therefore, it is urgent to address these challenges in order to increase the therapeutic effects of CAR-T cells targeting solid tumors in clinical settings.

CAR-T-loaded biomaterials facilitate the targeted accumulation of CAR-T cells at solid tumor sites, thereby significantly enhancing the therapeutic effects of CAR-T cells on the tumor. These biomaterials could be engineered to carry bioactive and chemical agents, further promoting CAR-T cell activation and proliferation. CAR-T cell-loaded biomaterials have been developed, from nickel alloy scaffolds to polysaccharide-degradable scaffolds. Compared to nickel alloy scaffolds, polysaccharide-degradable scaffolds avoid the need for a second operation in clinical settings. Recently, novel injectable hydrogels and microneedles have been developed to load CAR-T cells. These biomaterials can further reduce surgical trauma and are gradually degraded *in vivo*. Although the progress of biomaterials in enhancing CAR-T immunotherapy is promising, it is crucial to address concerns related to safety and stability when using these biomaterials in patients. Many biomaterials, such as microneedles and DNA scaffolds, involve complex preparation processes, making it challenging to meet the quality standards advised by the FDA for clinical-scale production. Therefore, simplifying and standardizing the biomaterial preparation process is essential.

Biological agents, such as chemokines and cytokines, play a vital role in modulating the infiltration, activity, and CAR-T cells of the immune system. By loading these biological agents in biomaterials designed to inhibit PD-1 and CTLA-4 to some extent, it was possible to reverse the tumor microenvironment (TME) and further trigger the endogenous immune response. Consequently, the controlled release of biological agents could activate specific signaling pathways, promoting CAR-T cell proliferation, activation, and infiltration. The released chemokines could also recruit immune cells to trigger an endogenous immune response, ultimately removing cancer cells. Given that the incidence rate of solid tumors is approximately 20 times higher than hematologic tumors, this presents a desired market opportunity for CAR-T therapy in solid tumors. Thus, the application of biomaterials has the potential to make significant breakthroughs for CAR-T therapy in solid tumors in clinical settings.

## References

[B1] Abigail K. G. Louai L. Dorota D. K. Gillie A. R. Peng X. Omokolade A. (2022). Delivery of CAR-T cells in a transient injectable stimulatory hydrogel niche improves treatment of solid tumors. Sci. Adv. 8 (14), eabn8264. 10.1126/sciadv.abn8264 35394838 PMC8993118

[B2] Adachi K. Kano Y. Nagai T. Okuyama N. Sakoda Y. Tamada K. (2018). IL-7 and CCL19 expression in CAR-T cells improves immune cell infiltration and CAR-T cell survival in the tumor. Nat. Biotechnol. 36, 346–351. 10.1038/nbt.4086 29505028

[B3] Agarwalla P. Ogunnaike E. A. Ahn S. Froehlich K. A. Jansson A. (2022). Bioinstructive implantable scaffolds for rapid *in vivo* manufacture and release of CAR-T cells. Nat. Biotech. 40, 1250–1258. 10.1038/s41587-022-01245-x PMC9376243 35332339

[B4] Andersen T. Auk-Emblem P. Dornish M. (2015). 3D cell culture in alginate hydrogels. Microarrays (Basel) 4, 133–161. 10.3390/microarrays4020133 27600217 PMC4996398

[B5] Anisha B. S. Vera J. E. Vinh X. T. Richard L. B. John S. F. Boyd N. R. (2022). Micro-hydrogel injectables that deliver effective CAR-T immunotherapy against 3D solid tumor spheroids. Transl. Oncol. 24, 101477. 10.1016/j.tranon.2022.101477 35905640 PMC9334344

[B6] Beatty G. L. Haas A. R. Maus M. V. Torigian D. A. Soulen M. C. Plesa G. (2014). Mesothelin-specific chimeric antigen receptor mRNA-engineered T cells induce anti-tumor activity in solid malignancies. Cancer Immunol. Res. 2, 112–120. 10.1158/2326-6066.cir-13-0170 24579088 PMC3932715

[B7] Bencherif S. A. Braschler T. M. Renaud P. (2013). Advances in the design of macroporous polymer scaffolds for potential applications in dentistry. J. Periodontal Implant Sci. 43 (6), 251–261. 10.5051/jpis.2013.43.6.251 24455437 PMC3891856

[B8] Bertrand N. Wu J. Xu X. Kamaly N. Farokhzad O. C. (2014). Cancer nanotechnology: the impact of passive and active targeting in the era of modern cancer biology. Clin. Cancer Res. 66, 22–25. 10.1016/j.addr.2013.11.009 PMC4219254 24270007

[B9] Bridges A. W. García A. J. (2008). Anti-inflammatory polymeric coatings for implantable biomaterials and devices. J. Diabetes Sci. Technol. 2 (6), 984–994. 10.1177/193229680800200628 19885288 PMC2769825

[B10] Brown C. Alizadeh D. Starr R. Weng L. Wagner J. R. Naranjo A. (2016). Regression of glioblastoma after chimeric antigen receptor T-cell therapy. N. Engl. J. Med. 375, 2561–2569. 10.1056/NEJMoa1610497 28029927 PMC5390684

[B11] Brown C. E. Warden C. D. Starr R. Deng X. Badie B. Yuan Y. C. (2013). Glioma IL13Rα2 is associated with mesenchymal signature gene expression and poor patient prognosis. PloS One 8 (10), e77769. 10.1371/journal.pone.0077769 24204956 PMC3800130

[B12] Burkhardt J. K. Carrizosa E. Shaffer M. H. (2008). The actin cytoskeleton in T cell activation. Annu. Rev Immunol 26, 233–259. 10.1146/annurev.immunol.26.021607.090347 18304005

[B13] Chang L. Chang W. Mcnamara G. Aguilar B. Ostberg J. Jensen M. (2007). Transgene-enforced co-stimulation of CD4+ T cells leads to enhanced and sustained anti-tumor effector functioning. Cytotherapy 9 (8), 771–784. 10.1080/14653240701656079 17917884

[B14] Chen Q. Hu Q. Elena D. Chen G. Ahn S. Wang C. (2019a). Photothermal therapy promotes tumor infiltration and antitumor activity of CAR T cells. Adv. Mater 3, 1900192. 10.1002/adma.201900192 PMC7262962 30916367

[B15] Chen Q. Wang C. Zhang X. Chen G. Hu Q. Li H. (2019b). *In situ* sprayed bioresponsive immunotherapeutic gel for post-surgical cancer treatment. Nat. Nanotechnol. 14, 89–97. 10.1038/s41565-018-0319-4 30531990

[B16] Cheung A. S. Zhang D. K. Y. Koshy S. T. Mooney D. J. (2018). Scaffolds that mimic antigen-presenting cells enable *ex vivo* expansion of primary T cells. Nat. Biotechnol. 36 (2), 160–169. 10.1038/nbt.4047 29334370 PMC5801009

[B17] Chmielewski M. Abken H. (2017). CAR T cells releasing IL-18 convert to T-Bethigh FoxO1low effectors that exhibit augmented activity against advanced solid tumors. Cell Rep. 21, 3205–3219. 10.1016/j.celrep.2017.11.063 29241547

[B18] Christopher T. M. Sandeep P. Yevgeny B (2020). Click cross-linking improves retention and targeting of refillable alginate depots. Acta Biomater. 112, 112–121. 10.1016/j.actbio.2020.05.033 32497743 PMC7365769

[B19] Coon M. E. Stephan S. B. Gupta V. Kealey C. P. (2020). Nitinol thin films functionalized with CAR-T cells for the treatment of solid tumours. Nat. Biomed. Eng. 4, 195–206. 10.1038/s41551-019-0486-0 31819155

[B20] Depil S. Duchateau P. Grupp S. A. Mufti G. Poirot L. (2020). 'Off-the-shelf' allogeneic CAR-T cells: development and challenges. Nat. Rev. Drug Discov. 19, 185–199. 10.1038/s41573-019-0051-2 31900462

[B21] Ding W. Zhou J. Zeng Y. Wang Y. n. Shi B. (2017). Preparation of oxidized sodium alginate with different molecular weights and its application for crosslinking collagen fiber. Carbohyd Polym. 157, 1650–1656. 10.1016/j.carbpol.2016.11.045 27987879

[B22] Dunn Z. S. Mac J. Wang P. T. (2019). T cell immunotherapy enhanced by designer biomaterials. Biomaterials 217, 119265. 10.1016/j.biomaterials.2019.119265 31271861 PMC6689323

[B23] Feng K. Liu Y. Guo Y. Qiu J. Wu Z. Dai H. (2018). Phase I study of chimeric antigen receptor modified T cells intreating HER2-positive advanced biliary tract cancers and pancreatic cancers. Protein Cell 9 (10), 838–847. 10.1007/s13238-017-0440-4 28710747 PMC6160389

[B24] Gloria B. K. Aragon-Sanabriaa V. Randolpha L. Jianga H. Joshua A. R. Becky S. W. (2020). High-affinity mutant Interleukin-13 targeted CAR T cells enhance delivery of clickable biodegradable fluorescent nanoparticles to glioblastoma. Bioact. Mater 5, 624–635. 10.1016/j.bioactmat.2020.04.011 32405577 PMC7212185

[B25] Han B. Song Y. Park J. Doh J. (2022). Nanomaterials to improve cancer immunotherapy based on *ex vivo* engineered T cells and NK cells. J. Control Release 343, 379–391. 10.1016/j.jconrel.2022.01.049 35124129

[B26] Hao M. Hou S. Li W. Li K. Xue L. Hu Q. (2020). Combination of metabolic intervention and T cell therapy enhances solid tumor immunotherapy. Sci. Transl. Med. 12, eaaz6667. 10.1126/scitranslmed.aaz6667 33239389

[B27] Hay K. A. Turtle C. J. (2017). Chimeric antigen receptor (CAR) T cells: lessons learned from targeting of CD19 in B-cell malignancies. Drugs 77, 237–245. 10.1007/s40265-017-0690-8 28110394 PMC5603178

[B28] Hegde M. Mukherjee M. Grada Z. Pignata A. Landi D. Navai S. A. (2016). Tandem CAR T cells targeting HER2 and IL13Rα2 mitigate tumor antigen escape. J. Clin. Invest 126 (8), 3036–3052. 10.1172/JCI83416 27427982 PMC4966331

[B29] Hu Q. Li H. Archibong E. Chen Q. Ruan H. Ahn S. (2021). Inhibition of post-surgery tumour recurrence via a hydrogel releasing CAR-T cells and anti-PDL1-conjugated platelets. Nat. Biomed. Eng. 5, 1038–1047. 10.1038/s41551-021-00712-1 33903744 PMC9102991

[B30] Hurton L. V. Singh H. Najjar A. M. Switzer K. C. Mi T. Maiti S. (2016). Tethered IL-15 augments antitumor activity and promotes a stem-cell memory subset in tumor-specific T cells. Proc. Natl. Acad. Sci. 113 (48), E7788–E7797. 10.1073/pnas.1610544113 27849617 PMC5137758

[B31] Hwang C. M. Sant S. Masaeli M. Kachouie N. N. Zamanian B. Lee S. H. (2010). Fabrication of three-dimensional porous cell-laden hydrogel for tissue engineering. Biofabrication 2, 035003. 10.1088/1758-5082/2/3/035003 20823504 PMC3282162

[B32] Ian C. M. Ali Z. Sun L. Phuengkham H. Adrian M. H. Gamboa L. (2021). Enhanced intratumoural activity of CAR T cells engineered to produce immunomodulators under photothermal control. Nat. Biomed. Eng. 5, 1348–1359. 10.1038/s41551-021-00781-2 34385695 PMC8791016

[B33] Jing J. Duo M. Jie C. Feng P. Yang P. (2022). Customized multifunctional peptide hydrogel scaffolds for CAR-T cell rapid proliferation and solid tumor immunotherapy. ACS Appl. Mater Interfaces 14, 37514–37527. 10.1021/acsami.2c10727 35944246

[B34] Katz S. C. Burga R. A. McCormack E. Wang L. Mooring W. Point G. R. (2015). Phase I hepatic immunotherapy for metastases study of intra-arterial chimeric antigen receptor-modified T-cell therapy for CEA+ liver metastases. Clin. Cancer Res. 21, 3149–3159. 10.1158/1078-0432.ccr-14-1421 25850950 PMC4506253

[B35] Kearney C. J. Mooney D. J. (2013). Macroscale delivery systems for molecular and cellular payloads. Nat. Mater 12 (11), 1004–1017. 10.1038/nmat3758 24150418

[B36] Kim J. Li W. A. Choi Y. Lewin S. A. Verbeke C. S. Dranoff G. (2015). Injectable, spontaneously assembling, inorganic scaffolds modulate immune cells *in vivo* and increase vaccine efficacy. Nat. Biotechnol. 33 (1), 64–72. 10.1038/nbt.3071 25485616 PMC4318563

[B37] Kofler D. M. Chmielewski M. Rappl G. Hombach A. Riet T. Schmidt A. (2011). CD28 costimulation impairs the efficacy of a redirected t-cell antitumor attack in the presence of regulatory T cells which can be overcome by preventing Lck activation. Mol. Ther. 19 (4), 760–767. 10.1038/mt.2011.9 21326215 PMC3070115

[B38] Kuo C. Ma P. (2001). Ionically crosslinked alginate hydrogels as scaffolds for tissue engineering: part1. Structure, gelation rate and mechanical properties. Biomaterials 22 (6), 511–521. 10.1016/S0142-9612(00)00201-5 11219714

[B39] Lee K. Y. Mooney D. J. (2012). Alginate: properties and biomedical applications. Prog. Polym. Sci. 37, 106–126. 10.1016/j.progpolymsci.2011.06.003 22125349 PMC3223967

[B40] Li H. Wang Z. Edikan A. O. Wu Q. Chen G. Hu Q. (2021). Scattered seeding of CAR T cells in solid tumors augments anticancer efficacy. Natl. Sci. Rev. 9 (3), nwab172. 10.1093/nsr/nwab172 35265340 PMC8900686

[B41] Liu F. Lin L. Zhang Y. Wang Y. Sheng S. Xu C. (2019). A tumor-microenvironment-activated nanozyme-mediated theranostic nanoreactor for imaging-guided combined tumor therapy. Adv. Mater 31, 1902885. 10.1002/adma.201902885 31423690

[B42] Long A. H. Haso W. M. Shern J. F. Wanhainen K. M. Murgai M. Ingaramo M. (2015). 4-1BB costimulation ameliorates T cell exhaustion induced by tonic signaling of chimeric antigen receptors. Nat. Med. 21 (6), 581–590. 10.1038/nm.3838 25939063 PMC4458184

[B43] Luo Y. Chen Z. Sun M. Li B. Pan F. Ma A. (2022). IL-12 nanochaperone-engineered CAR T cell for robust tumor-immunotherapy. Biomaterials 281, 121341. 10.1016/j.biomaterials.2021.121341 34995901

[B45] Luo Z. Liu Z. Liang Z. Pan J. Xu J. Dong J. (2020). Injectable porous microchips with oxygen reservoirs and an immune-niche enhance the efficacy of CAR T cell therapy in solid tumors. ACS Appl. Mater Interfaces 12 (51), 56712–56722. 10.1021/acsami.0c15239 33306365

[B46] Ma X. Shou P. Smith C. Chen Y. Du H. Sun C. (2020). Interleukin-23 engineering improves CAR T cell function in solid tumors. Nat. Biotechnol. 38, 448–459. 10.1038/s41587-019-0398-2 32015548 PMC7466194

[B47] Madelyn V. Pritha A Sharda P. Yevgeny B. (2022). Fabrication and use of dry macroporous alginate scaffolds for viral transduction of T Cells. J. Vis. Exp. 187, e36156536. 10.3791/64036 36156536

[B48] Nayak A. K. Mohanta B. C. Hasnain M. S. Hoda M. N. Tripathi G. (2020). Chapter 14-Alginate-based scaffolds for drug delivery in tissue engineering. Alginates Drug Deliv., 359–386. 10.1016/B978-0-12-817640-5.00014-5

[B49] Neelapu S. S. Locke F. L. Bartlett N. L. Lekakis L. J. Reagan P. M. Miklos D. B. (2021). Comparison of 2-year outcomes with CAR T cells (ZUMA-1) vs salvage chemotherapy in refractory large B-cell lymphoma. Blood Adv. 5, 4149–4155. 10.1182/bloodadvances.2020003848 34478487 PMC8945634

[B50] Pritha A. Edikan A. O. Ahn S. Kristen A. F. Jansson A. Frances S. L. (2022). Bioinstructive implantable scaffolds for rapid *in vivo* manufacture and release of CAR-T cells. Nat. Biotechnol. 40, 1250–1258. 10.1038/s41587-022-01245-x 35332339 PMC9376243

[B51] Savoldo B. Ramos C. A. Liu E. Mims M. P. Keating M. J. Carrum G. (2011). CD28 costimulation improves expansion and persistence of chimeric antigen receptor-modified T cells in lymphoma patients. J. Clin. Invest 121, 1822–1826. 10.1172/JCI46110 21540550 PMC3083795

[B52] Schmid D. Park C. G. Hartl C. A. Subedi N. Cartwright A. N. Puerto R. B. (2017). T cell-targeting nanoparticles focus delivery of immunotherapy to improve antitumor immunity. Nat. Commun. 8, 1747. 10.1038/s41467-017-01830-8 29170511 PMC5700944

[B53] Shi D. Shi Y. Kaseb A. O. Qi X. Zhang Y. Chi J. (2020). Chimeric antigen receptor-glypican-3 T-cell therapy foradvanced hepatocellular carcinoma: results of phase I trials. Clin. Cancer Res. 26 (15), 3979–3989. 10.1158/1078-0432.CCR-19-3259 32371538

[B54] Smith T. T. Moffett H. F. Stephan S. B. Opel C. F. Dumigan A. G. Jiang X. (2017). Biopolymers codelivering engineered T cells and STING agonists can eliminate heterogeneous tumors. J. Clin. Invest 127, 2176–2191. 10.1172/JCI87624 28436934 PMC5451231

[B55] Stephan S. B. Taber A. M. Jileaeva I. Pegues E. P. Sentman C. L. Stephan M. T. (2015). Biopolymer implants enhance the efficacy of adoptive T-cell therapy. Nat. Biotechnol. 33 (1), 97–101. 10.1038/nbt.3104 25503382 PMC4289408

[B56] Tang L. Zheng Y. Melo M. B. Mabardi L. Castano A. P. Xie Y. Q. (2018). Enhancing T cell therapy through TCR-signaling-responsive nanoparticle drug delivery. Nat. Biotechnol. 36, 707–716. 10.1038/nbt.4181 29985479 PMC6078803

[B57] Tang Y. Yao W. Hu H. Xiong W. Mei H. Hu Y. (2023). TGF-β blocking combined with photothermal therapy promote tumor targeted migration and long-term antitumor activity of CAR-T cells. Mater Today Bio 20, 100615. 10.1016/j.mtbio.2023.100615 PMC10090704 37063775

[B58] Tchou J. Zhao Y. Levine B. L. Zhang P. Davis M. M. Melenhorst J. J. (2017). Safety and efficacy of intratumoral injections of chimeric antigen receptor (CAR) T cells in metastatic breast cancer. Cancer Immunol. Res. 5, 1152–1161. 10.1158/2326-6066.CIR-17-0189 29109077 PMC5712264

[B59] Tsao C. T. Kievit F. M. Ravanpay A. Erickson A. E. Jensen M. C. Ellenbogen R. G. (2014). Thermoreversible poly(ethylene glycol)-g-chitosan hydrogel as a therapeutic T lymphocyte depot for localized glioblastoma immunotherapy. Biomacromolecules 15 (7), 2656–2662. 10.1021/bm500502n 24890220 PMC4215871

[B60] Van M. C. Papa S. E. Jeannon J. P. Urbano T. G. Spicer J. F. Maher J. (2013). Design of a phase I clinical trial to evaluate intratumoral delivery of ErbB-targeted chimeric antigen receptor T-cells in locally advanced or recurrent head and neck cancer. Hum. Gene Ther. Cl. Dev. 24 (3), 134–142. 10.1089/humc.2013.144 24099518

[B61] Verbeke C. S. Mooney D. J. (2015). Injectable, pore-forming hydrogels for *in vivo* enrichment of immature dendritic cells. Adv. Healthc. Mater 4 (17), 2677–2687. 10.1002/adhm.201500618 26474318 PMC4715727

[B62] Villa G. R. Mischel P. S. (2016). Old player, new partner: EGFRvIII and cytokine receptor signaling in glioblastoma. Nat. Neurosci. 19 (6), 765–767. 10.1038/nn.4302 27227363

[B63] Wang C. M. Wu Z. Q. Wang Y. Guo Y. L. Dai H. R. Wang X. H. (2017). Autologous T cells expressing CD30 chimeric antigen receptors for relapsed or refractory hodgkin lymphoma: an open-label phase I trial. Clin. Cancer Res. 23, 1156–1166. 10.1158/1078-0432.CCR-16-1365 27582488

[B64] Wang K. Chen Y. Ahn S. Zheng M. Landoni E. Dotti G. (2020). GD2-specific CAR T cells encapsulated in an injectable hydrogel control retinoblastoma and preserve vision. Nat. Cancer 1, 990–997. 10.1038/s43018-020-00119-y 33898999 PMC8061756

[B65] Wen Y. Waltman A. Han H. Collier J. H. (2016). Switching the immunogenicity of peptide assemblies using surface properties. ACS Nano 10 (10), 9274–9286. 10.1021/acsnano.6b03409 27680575 PMC5704984

[B66] Xiao H. Jasper Z. W. Ryan C. Li Z. Burnett C. E. Hernandez-Lopez R. (2021). DNA scaffolds enable efficient and tunable functionalization of biomaterials for immune cell modulation. Nat. Nanotechnol. 16, 214–223. 10.1038/s41565-020-00813-z 33318641 PMC7878327

[B67] Xu B. Cui Y. Wang W. Li S. Lyu C. Wang S. (2020). Immunomodulation-enhanced nanozyme-based tumor catalytic therapy. Adv. Mater 32, 2003563. 10.1002/adma.202003563 32627937

[B68] Xu J. Wang Y. Shi J. Liu J. Li Q. Chen L. (2018). Combination therapy: a feasibility strategy for CAR-T cell therapy in the treatment of solid tumors. Oncol. Lett. 16, 2063–2070. 10.3892/ol.2018.8946 30008901 PMC6036511

[B69] Xue Y. Che J. Ji X. Li Y. Xie J. Chen X. (2022). Recent advances in biomaterial-boosted adoptive cell therapy. Chem. Soc. Rev. 51, 1766–1794. 10.1039/D1CS00786F 35170589

[B70] Yang B. Chen Y. Shi J. (2019). Nanocatalytic medicine. Adv. Mater 31, 1901778. 10.1002/adma.201901778 31328844

[B71] Yi Z. Prinzing B. L. Cao F. Gottschalk S. Krenciute G. (2018). Optimizing EphA2-CAR T cells for the adoptive immunotherapy of glioma. Mol. Ther-Meth Clin. D. 9, 70–80. 10.1016/j.omtm.2018.01.009 PMC5852415 29552579

[B72] Yong C. S. Dardalhon V. Devaud C. Taylor N. Darcy P. K. Kershaw M. H. (2017). CAR T-cell therapy of solid tumors. Immunol. Cell Biol. 95, 356–363. 10.1038/icb.2016.128 28003642

[B73] Zheng H. Wu Q. Li H. J. Gu Z. (2022). Integration of synthetic biology and nanobiotechnology for biomedical applications. Synth. Biol. J. 3, 279–301. 10.12211/2096-8280.2022-008

[B74] Zhu L. Gao D. Xie L. Dai Y. Zhao Q. (2020). NIR II-excited and pH-responsive ultrasmall nanoplatform for deep optical tissue and drug delivery penetration and effective cancer chemophototherapy. Mol. Pharm. 17, 3720–3729. 10.1021/acs.molpharmaceut.0c00404 32633977

[B75] Zhu L. Liu J. Zhou G. Liu T. Dai Y. Nie G. (2021). Remodeling of tumor microenvironment by tumor targeting nanozymes enhances immune activation of CAR-T cells for combination therapy. Small 17 (43), e2102624. 10.1002/smll.202102624 34378338

